# Agonistic Interactions in Pigs–Comparison of Dominance Indices with Parameters Derived from Social Network Analysis in Three Age Groups

**DOI:** 10.3390/ani9110929

**Published:** 2019-11-07

**Authors:** Kathrin Büttner, Irena Czycholl, Katharina Mees, Joachim Krieter

**Affiliations:** Institute of Animal Breeding and Husbandry, Christian-Albrechts-University, Olshausenstr. 40, D-24098 Kiel, Germany

**Keywords:** agonistic interaction, pig, dominance indices, centrality parameters, social network analysis

## Abstract

**Simple Summary:**

The importance of animals within a social group can be ranked with the aid of centrality parameters, e.g., measures derived from social network analysis. In the present study, it was investigated whether these centrality parameters capture a similar rank order compared to dominance indices which are calculated based on the number of won and lost fights. Social networks for animals in three repeated mixing events were built (weaned piglets, fattening pigs, gilts) based on different types of interactions (in the present study, initiating and receiving agonistic interactions, and winning or losing a fight). Centrality parameters based on active behavior, especially winning an agonistic interaction, showed a similar rank order compared to the dominance indices. Also, the results of partial least squares structural equation modelling showed that the networks built on information about winning or losing a fight could best illustrate the dominance structure with an explained variance of about 60% for all three age groups. Thus, network analysis can provide information about the dominance structure within the group and also has the advantage of including indirect relationships between the animals which cannot be supported by the dyadic approach.

**Abstract:**

Dominance indices are often calculated using the number of won and lost fights of each animal focusing on dyadic interactions. Social network analysis provides new insights into the establishment of stable group structures going beyond the dyadic approach. Thus, it was investigated whether centrality parameters describing the importance of each animal for the network are able to capture the rank order calculated by dominance indices. Therefore, two dominance indices and five centrality parameters based on two network types (initiator-receiver and winner-loser networks) were calculated regarding agonistic interactions observed in three mixing events (weaned piglets, fattening pigs, gilts). Comparing the two network types, the winner-loser networks demonstrated highly positive correlation coefficients between out-degree and outgoing closeness and the dominance indices. These results were confirmed by partial least squares structural equation modelling (PLS-SEM), i.e., about 60% of the variance of the dominance could be explained by the centrality parameters, whereby the winner-loser networks could better illustrate the dominance hierarchy with path coefficients of about 1.1 for all age groups. Thus, centrality parameters can portray the dominance hierarchy providing more detailed insights into group structure which goes beyond the dyadic approach.

## 1. Introduction

Repeated rehousing and mixing events with new group compositions are common practice in pig husbandry. This standard procedure leads to fights between the animals which try to thereby establish a new stable social hierarchy within the group. In this context, a variety of individual pig behavior is shown ranging from subtle ritualized displays to overt agonistic interactions with high intensity of aggression [1].

In domestic pigs, the establishment of a stable social hierarchy is mainly based on overt agonistic interactions and can lead to injuries (e.g., skin lesions) and then negatively impact animal health, welfare and production parameters [2,3].

Captive animals, which are housed in an artificial environment with restricted available space and means of escape as well as predefined pen mates, are to a lesser extent able to perform natural behavior or to follow the common behavioral rules (e.g., avoidance distance), which are necessary to establish a stable group structure. Farm animals are kept regardless of their inherent social behavior possibly leading to increased agonistic interactions or behavioral problems [4]. Thus, deeper insight into the formation of stable group structures and how animals behave in rehousing and mixing events can be used for managing aggression and to implement or optimize strategies for the reduction of agonistic interactions [5].

A stable group structure is achieved when all animals have their individual rank position within the group. In order to describe these rank positions, so-called dominance indices were defined which help to rank the animals within a group from dominant to subordinate individuals. These dominance indices are usually calculated with the help of the outcome of agonistic interactions for dyadic relationships. For each pair of animals within a group the number of won and lost fights is calculated per animal.

However, these dominance indices are based on dyadic interactions and do not take the whole network structure into account. Another possible approach is to characterize the social structures within a group using social network analysis. Here, not only the pairwise interactions between the animals can be analyzed but also the whole group structure as well as indirect relationships between the animals are considered [6]. In a network, the animals are so-called nodes and the links between them are represented as so-called edges. These edges can represent different kinds of interactions, such as agonistic interactions, grooming, food competition or sharing the same local area. In the case of agonistic interactions, these edges can, for example, be interpreted as the relation between initiated and received fights pointing from the initiator of an agonistic interaction to the receiver or the relation between won and lost fights pointing from the winner of a fight to the loser. Due to the fact that each edge has a clear direction the whole network is considered directed. Social network analysis offers a huge tool box of standardized methods to calculate network and centrality parameters focusing on global descriptors of the entire network as well as on the importance of individual nodes for the whole group structure [7,8,9]. Centrality parameters help to range the animals in their position within the group, i.e., the animals are ranked according to their importance for the network. Depending on the calculated centrality parameter these ranks are based on different characteristics, e.g., direct contacts (in-degree and out-degree), direct as well as indirect contacts (ingoing and outgoing closeness) or shortest paths (betweenness), which give different interpretations for the importance of the individual animal for the group structure. One particular advantage of social network analysis over the dyadic approach is that the animals are treated as interdependent elements which are all connected within the network, which accounts for the fact that the behavior of one individual in the group can affect the behavior of its pen mates [10]. Understanding the complex group structure and the importance or centrality of each individual within this group has essential implications for applied animal behavior and welfare research [5].

Although the social network approach provides new insights into the formation and development of animal behavior regarding management and welfare aspects, the application of social network analysis for farm animals is still underrepresented (e.g., agonistic interactions in pigs [6,11,12], dynamic groups structure in dairy cows [13,14], tail biting in pigs [15]).

The aim of the present study was to answer the question of whether rank positions obtained from centrality parameters are able to portray the same social rank obtained from dominance indices. For this purpose, in three rehousing and mixing events covering different age groups (weaned piglets, fattening pigs, gilts), agonistic interactions were recorded in order to calculate dominance indices as well as to perform social network analysis. If specific centrality parameters are able to capture the social rank of the animals, these parameters can be used for future studies focusing on dominance structures and additionally providing the whole advantages of network analysis which goes beyond the simple dyadic approach.

## 2. Materials and Methods 

### 2.1. Animals and Housing

The study was carried out on the research farm “Hohenschulen” of the Institute of Animal Breeding and Husbandry of the University of Kiel, Germany, from December 2010 until August 2012. The herd consisted of purebred and crossbred animals of the German Landrace and Large White breeds. All the males were castrated. In each rehousing and mixing event, the animals were sorted by the smallest level of familiarity, i.e., the number of animals acquainted with each other from former pens was minimized. In the present study, agonistic behavior was analyzed in three different rehousing and mixing events (weaned piglets, fattening pigs, gilts). The age of the animals at rehousing and mixing, the maximum level of familiarity from previous mixing events, the housing conditions, the number of animals included in the study as well as the analyzed observation periods are presented in Table 1.

### 2.2. Recording and Analysis of Agonistic Interactions

In order to continuously record the behavior of the animals, video cameras were mounted on the ceiling of each pen, which enabled a complete overview of all animals. For the clear identification of initiator and receiver as well as winner and loser of an agonistic interaction, each animal was marked individually on its back using livestock marking spray. The study of Stukenborg et al. [16] stated a decline in fighting behavior during the night. Therefore, only the daytime records were analyzed in the present study. Furthermore, other studies have stated a reduction in agonistic interactions in the whole group 48 h after mixing, whereby the animals with the highest rank obtained their positions within the first hour and other high ranking animals within 4 to 6 h after mixing [17]. Moreover, Friend et al. [18] stated a reduction in agonistic interactions within 3 h after mixing. Thus, in the present study, 2.5 days (28 h) for weaned piglets and 1.5 days (17 h) for fattening pigs and gilts were analyzed. The shorter observation period for fattening pigs and gilts was chosen due to the fact that these two older age groups revealed a clear drop in fighting frequency within 17 h of video observation. However, in order to enable a comparison between weaned piglets and the two older age groups, the results for weaned piglets were presented for 17 h as well as 28 h of video observation. Video recording started directly after rehousing and mixing at 12:00 h on day 1. The observation periods for each age group are illustrated in Table 1 and were analyzed by three observers for weaned piglets and one observer for fattening pigs and gilts using the HeiTelPlayer software (Xtralis Headquarter D-A-CH, HeiTel Digital Video GmbH, Kiel, Germany). 

Before the video analysis started, training was carried out using unknown video sequences in order to ensure the correct definition and detection of agonistic interactions (interobserver reliability above 90%). An agonistic interaction was defined as fight or physical displacement by one pig towards another which lasted longer than 1 s and included aggressive behavior. This aggressive behavior (parallel/inverse parallel pressing, head to body knock, head to head knock, biting, physical displacement) was followed by submissive behavior (turning away, fleeing) by the loser of the agonistic interaction [19,20,21,22], which indicates the end of a decisive agonistic interaction when the pigs were separated for at least 5 s [23]. If no clear submissive behavior of one of the involved animals could be detected and the pigs were separated for at least 5 s, the outcome of the agonistic interaction was recorded as indecisive. For each agonistic interaction, the initiator and receiver, as well as winner and loser, were recorded. Only agonistic interactions with a clear initiator and receiver as well as a clear winner and loser were included in the further analysis. For weaned piglets, after 17 h or 28 h of video observation 5088 or 7620 agonistic interactions, respectively, in 93 pens were recorded, which corresponds to 12.3 ± 11.1 or 18.4 ± 17.6 agonistic interactions/animal. For fattening pigs and gilts, after 17 h of video observation 1611 agonistic interactions in 26 pens (5.9 ± 4.6 agonistic interactions/animal) and 665 agonistic interactions in 12 pens (5.3 ± 4.6 agonistic interactions/animal) were recorded.

### 2.3. Dominance Indices

In order to obtain the hierarchical position of individual animals in a group, different dominance indices can be calculated.

The dominance index according to Bowen and Brooks [24] ($DI_{1}$) is measured with the following formula:

(1) $$DI_{1}=\frac{wins-defeats}{wins+defeats}$$

The dominance index used in Borberg and Hoy [25] and Fels et al. [26] ($DI_{2}$) includes additionally to the number of won and lost fights (wins, defeats), the number of pen mates the pig had won ($P_{won}$) or lost ($P_{lost}$) against and the group size (n):

(2) $$DI_{2}=\;\frac{\left(wins\times P_{won}\right)-\left(defeats\times P_{lost}\right)}{\left(wins+defeats\right)\times \left(n-1\right)}$$

Both dominance indices can only be calculated for animals which were involved in at least one agonistic interaction. The values range between −1 (absolute subordinate animal) and +1 (absolute dominant animal). 

### 2.4. Network Construction and Centrality Parameters

**Figure 1:**
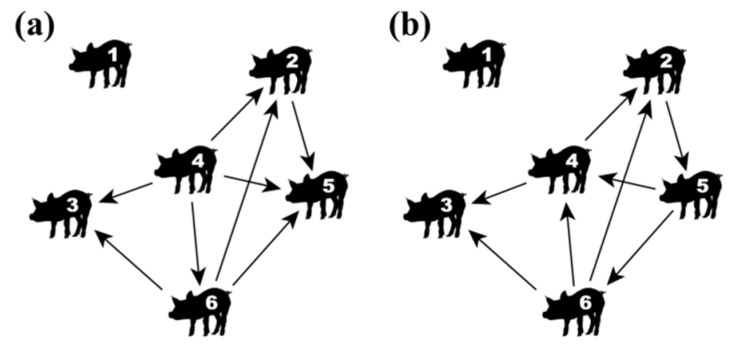
Example illustration of (**a**) an initiator-receiver network and (**b**) a winner-loser network within the same group of animals. In the initiator-receiver network, the edges point from the initiator to the receiver of an agonistic interaction. In the winner-loser network, the edges point from the winner to the loser of the agonistic interaction.

Two types of social networks were created for each pen to comprise different viewpoints on agonistic interactions: initiator-receiver networks and winner-loser networks. The initiator-receiver networks contained directed edges between the animals, whenever one animal attacked another one, i.e., the network contained a directed edge pointing from the initiator to the receiver of an agonistic interaction. The winner-loser networks contained directed edges between the animals, whenever one animal won a fight against another one, i.e., the network contained a directed edge pointing from the winner to the loser of an agonistic interaction. Multiple edges between each pair of animals were aggregated to a single one. Through these edges, the animals are connected with each other by so-called paths, which represent either direct or indirect connections between the animals. In directed networks, each path has to follow the direction of edges. Figure 1 illustrates example networks for both network types. Here, pig 3 was attacked by pig 4 and pig 6 (Figure 1a) and lost both fights against its attackers (Figure 1b). In contrast, pig 4 initiated four agonistic interactions (Figure 1a), but only two of them (i.e., pig 4 vs. pig 2 and pig 4 vs. pig 3) resulted in a won fight for pig 4 (Figure 1b). Furthermore, pig 1 never participated in any agonistic interaction and is therefore isolated, i.e., there is no connection to at least one other animal. 

Centrality parameters help to range the animals in their central position within the group and enable the detection of key individuals which play an important role in the social structure of the entire network. A detailed description of the calculated centrality parameters is provided in Table 2. All centrality parameters were calculated using the Python module NetworkX [27]. 

### 2.5. Statistical Analysis

All statistical analyses were carried out using the SAS^®^ statistical software package 9.4 (SAS^®^ Institute Inc., Cary, NC, USA) [31].

For the evaluation of the temporal development of the dominance indices and the parameters derived from social network analysis, they were calculated using rising time window lengths over the whole observation period. This means, the first time window length encompassed the observation times day 1: 12:00 until day 1: 13:00 (amounts to 1 h), the second time window length the observation times day 1: 12:00 until day 1: 14:00 (amounts to 2 h) and so on until the entire observation period from day 1: 12:00 until day 3: 18:00 (amounts to 28 h) for weaned piglets or until day 2: 18:00 (amounts to 17 h) for fattening pigs and gilts was covered.

In order to evaluate the relation between the dominance indices and the centrality parameters for both network types in the temporal course, Spearman rank correlation coefficients ($r_{s}$) were calculated for each time window length. The $r_{s}$ was used as an evaluation criterion due to the fact that it does not directly compare the values obtained but their rank order [32]. For the present study, this ranking is of special importance as the question was whether the ordering of the animals considering dominance indices or centrality parameters stayed the same, or whether there was a shift within the ranking. The $r_{s}$ values range between −1 to 1, where, regarding the present research question, higher absolute values are preferred. To be more precise, a high correlation is assumed for absolute $r_{s}$ values equal to or greater than 0.7, a moderate correlation is assumed for absolute $r_{s}$ values ranging between 0.4 and 0.7 and poor correlation is assumed for absolute $r_{s}$ values ranging from 0 to 0.4 [32]. High negative $r_{s}$ values display a reversed order of the two parameters compared.

Besides the Spearman rank correlation, the rank differences between subsequent rank positions were also analyzed for all parameters. This means, the differences in standardized parameters between rank positions 1 and 2 (referred to as comparison 1:2), between rank positions 2 and 3 (referred to as comparison 2:3), and so on, were calculated for each individual pen. The impact of the calculated parameters ($DI_{1}$, $DI_{2}$, in-degree, out-degree, betweenness, ingoing closeness and outgoing closeness), the specific comparisons (1:2, 2:3, 3:4, …) as well as the interaction between the fixed effects parameter and comparison on the individual rank differences were tested using the MIXED procedure of the statistical software package SAS 9.4 [31]. Due to the low number of observations for the comparison of the lower-ranked animals, the model included the rank differences up to the comparison 8:9 for weaned piglets, up to the comparison 20:21 for fattening pigs and up to the comparison 18:19 for gilts. To determine significant differences between $DI_{1}$ and all other parameters only pairwise comparisons with $DI_{2}$ were calculated and adjusted by the Bonferroni–Holm correction [33].

Furthermore, partial least squares structural equation modelling (PLS-SEM) was carried out using the SmartPLS 3.0 software (SmartPLS GmbH, Boenningsted, Germany)) [34] to detect to what extent the centrality parameters were able to portray the dominance indices. This has the advantage of a more holistic approach. For this statistical analysis, the dataset comprised the whole observation period of 28 h for weaned piglets and 17 h for fattening pigs and gilts. Thus, a separated model was carried out for each age group. The models include latent variables that are not directly measurable (the endogenous variable dominance (D) and the exogenous variables centrality based on initiator-receiver networks (C_IR_) and centrality based on winner-loser networks (C_WL_)) and measurement variables which enable an estimation of the latent variables. In the present study, the measurement variables comprised the two dominance indices as well as the single centrality parameters calculated for the two network types. The PLS-SEM consists of two parts: the inner and the outer model. The inner model represents the relationships between the latent variables characterized by the so-called path coefficients and the coefficient of determination (R^2^) for the endogenous variable indicating to which extent the endogenous variable is explained by other latent variables. The outer model presents the impact of each measurement variable on the related latent variable characterized by the so-called indicator reliabilities. The goodness-of-fit of the PLS-SEM was performed using the quality criteria proposed by Hair et al. [35] in a two-stage process. Firstly, the inner models were tested including the internal consistency reliability (composite reliability) and convergent validity (indicator reliability and average variance extracted (AVE)). The composite reliability takes the different loadings of the indicators into account and illustrates values between 0 and 1. In exploratory studies, values between 0.6 and 0.9 should be assumed. The indicator reliability indicates how sufficient a latent variable is estimated by an indicator and should achieve values above 0.7. AVE describes the extent to which a latent variable explains the variance of its indicators and should achieve values above 0.5. Secondly, the outer model is tested including an examination of the coefficients of determination (R^2^) and the relevance and statistical significance of the path coefficients tested with bootstrapping. R^2^ values of 0.25, 0.50 and 0.75 are assessed as weak, moderate and substantial [35,36], whereby higher values are preferred. Path coefficients characterize the relationships between the latent variables. Similar to correlation, coefficients values close to 1 indicate a strong positive relationship and vice versa for negative values. Values close to 0 represent very weak relationships between the latent variables.

## 3. Results

### 3.1. Descriptive Statistics

#### 3.1.1. Number of Isolated Animals 

Considering the number of animals for which no dominance index or centrality parameter could be obtained, weaned piglets started with on average 54% isolated animals/pen, which showed a clear decrease within the first 6 h to 13%. This decrease proceeded further to 1% of the animals/pen after 17 h of video observation and to 0.4% of the animals/pen after 28 h of video observation. For these animals, no dominance index or centrality parameter could be obtained. For fattening pigs and gilts housed in larger groups, the number of isolated nodes started at a higher level with 83% and 78%. However, also in the higher age groups, a clear decrease was obvious within the first 6 h after rehousing and mixing to 12% for fattening pigs and 16% for gilts. At the end of the observation period, for 2% and 5%, respectively, of the animals/pen, no dominance index or centrality parameter could be calculated.

#### 3.1.2. Relation between Initiated and Won Fights

For weaned piglets, after 1 h of video observation, 56% of all initiated fights resulted in a victory for the initiator. This amount increased slightly to 66% and stayed constantly at this level for the whole observation period. For fattening pigs, the amount at the beginning of the observation period was lower, i.e., 26% of initiated fights resulted in a victory. This amount increased to 72% within 4 h, followed by a slight decrease over the rest of the observation period to 68%. For gilts, the overall number of initiated fights which resulted in a victory was slightly lower compared to the other age groups. Here, at the beginning of the observation period, 49% of all initiated fights resulted in a victory. This amount increased further to 54% over the whole observation period.

#### 3.1.3. Dominance Indices

**Figure 2:**
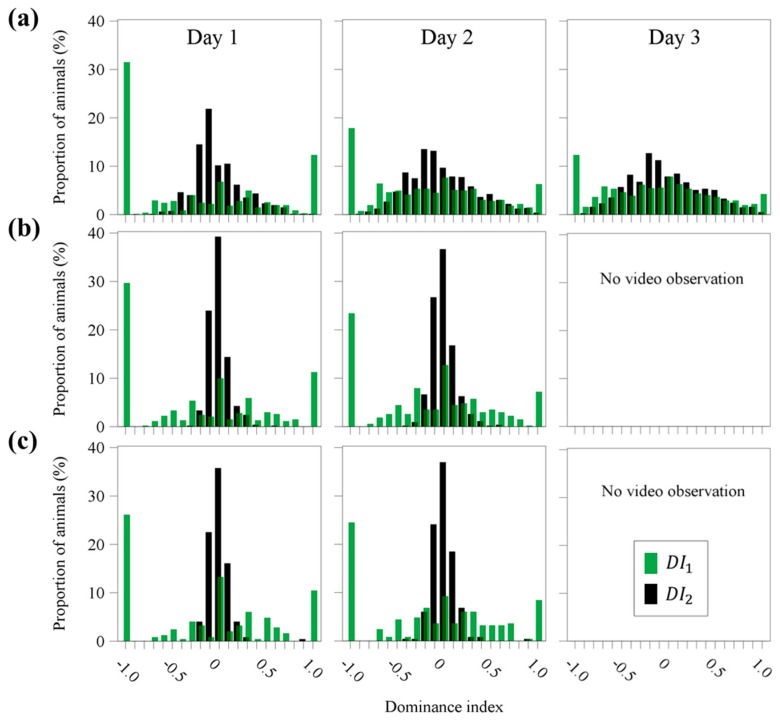
Frequency distributions of the dominance indices ($DI_{1}$ and $DI_{2}$ ) for the three age groups ((**a**) weaned piglets, (**b**) fattening pigs, (**c**) gilts) after 1, 2 or 3 days of video observation.

Figure 2 illustrates the frequency distributions of the dominance indices $DI_{1}$ and $DI_{2}$ for the three age groups after 1, 2 and 3 days of video observation. Comparing the two dominance indices it becomes obvious that $DI_{1}$ had a much wider range covering the whole interval of possible values from −1 to 1 compared to $DI_{2}$. In the older age groups, fattening pigs and gilts, the differences between $DI_{1}$ and $DI_{2}$ became more evident. Here, for $DI_{2}$ most of the animals had a dominance index between −0.2 and 0.2. However, although the frequency distribution between the two dominance indices differed from each other, the Spearman rank correlation coefficients between them showed high $r_{s}$ values ranging from 0.85 to 0.92 in the temporal course and for all age groups.

#### 3.1.4. Centrality Parameters

**Figure 3:**
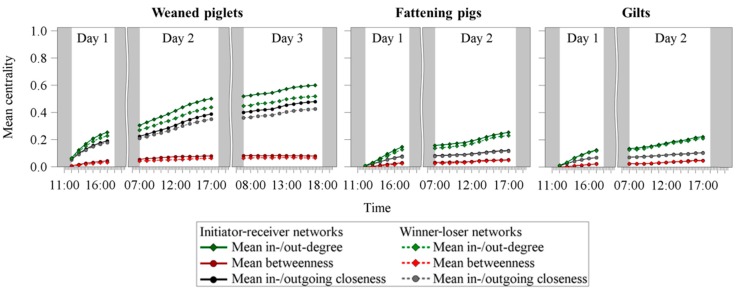
Temporal development of the mean centrality parameters for the initiator-receiver networks and the winner-loser networks for the age groups: weaned piglets, fattening pigs and gilts. For illustration purposes, the figure illustrates the standardized in- or out-degree centrality ranging from 0 to 1 in order to enhance the comparability to the other centrality parameters.

Figure 3 illustrates the temporal development of the mean centrality parameters for the initiator-receiver as well as the winner-loser networks considering the three age groups: weaned piglets, fattening pigs and gilts. In all three age groups, the mean in-/out-degree always revealed the highest values, followed by the mean in-/outgoing closeness and the betweenness. The two older age groups housed in larger groups demonstrated smaller differences between the centrality parameters of the initiator-receiver networks and the winner-loser networks, so that for gilts, nearly no differences between both network types could be obtained. 

### 3.2. Spearman Rank Correlation Coefficients between the Dominance Indices and the Centrality Parameters in the Temporal Course

**Figure 4:**
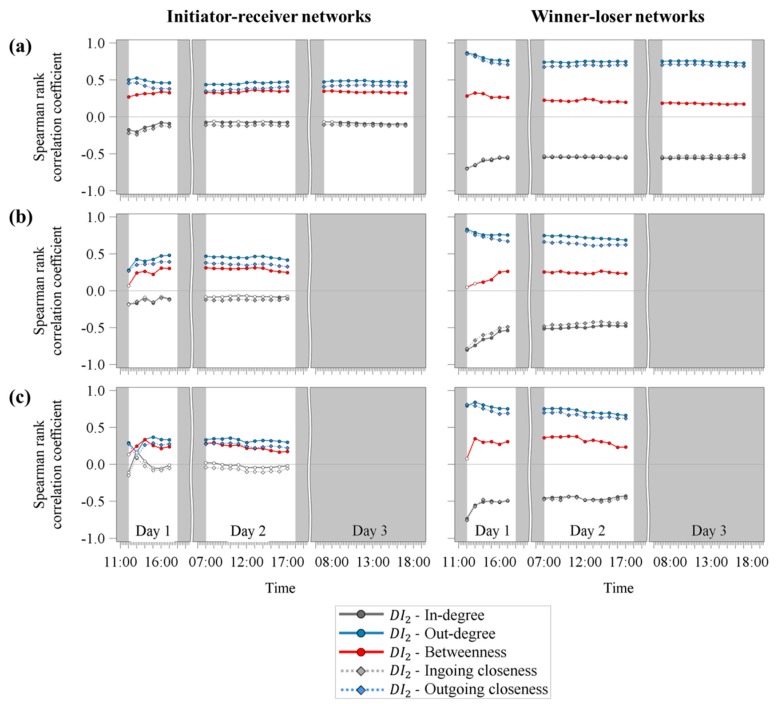
Temporal development of the Spearman rank correlation coefficients between the dominance index ($DI_{2}$) and the centrality parameters for (**a**) weaned piglets, (**b**) fattening pigs and (**c**) gilts. Filled markers indicate significant correlation coefficients (*p* < 0.05). Grey shaded areas are times without video observation.

Figure 4 illustrates the temporal course of the Spearman rank correlation coefficients between $DI_{2}$ and the centrality parameters for the initiator-receiver and the winner-loser networks separated by the three observed age groups: weaned piglets, fattening pigs and gilts. Comparing the results of the initiator-receiver networks and the winner-loser networks in all three observed age groups, the correlation coefficients could more easily be distinguished between the single centrality parameters in the winner-loser network compared to the initiator-receiver networks. In the winner-loser networks, three clearly distinguishable groups were obtained for all three age groups: highly positive $r_{s}$ values for out-degree and outgoing closeness, moderate negative $r_{s}$ values for in-degree and ingoing closeness and low to moderate positive $r_{s}$ values for betweenness.

For weaned piglets, $DI_{2}$ was highly correlated with out-degree (ranging from 0.73 to 0.87) and outgoing closeness (ranging from 0.68 to 0.85) in the winner-loser networks, whereas for the initiator-receiver networks the values ranged only from 0.43 to 0.53 for out-degree and 0.35 and 0.46 for outgoing closeness. A similar picture could be obtained for the correlation coefficients between $DI_{2}$ and the centrality parameters in-degree and ingoing closeness. In contrast to the centrality parameters focusing on outgoing interactions, in-degree and ingoing closeness were highly negatively correlated with $DI_{2}$ ranging from −0.54 to −0.70 or −0.51 to −0.70 for the winner-loser networks, whereas the $r_{s}$ values for the initiator-receiver network were only low to moderate ranging from −0.07 to −0.20 for in-degree and −0.1 to −0.23 for ingoing closeness. The $r_{s}$ values between $DI_{2}$ and betweenness revealed slightly higher values for the initiator-receiver networks ranging from 0.27 to 0.37 compared to the winner-loser networks ranging from 0.17 to 0.32.

A similar trend was observed for fattening pigs. For the winner-loser networks, the $r_{s}$ values between $DI_{2}$ and out-degree as well as outgoing closeness were the highest ranging from 0.69 to 0.83 and 0.61 to 0.81. $DI_{2}$ and in-degree, as well as ingoing closeness, showed moderate to highly negative $r_{s}$ values ranging from −0.47 to −0.80 or −0.42 to −0.78. Except for the correlation between $DI_{2}$ and betweenness, the values of the winner-loser networks revealed always higher correlation coefficients (positive or negative) compared to the initiator-receiver networks. 

Gilts showed similar results compared to the other two age groups with the exception of the correlation coefficients between $DI_{2}$ and the centrality parameters focusing on ingoing interactions, i.e., in-degree and ingoing closeness. Here, no significant $r_{s}$ values could be obtained. 

The temporal course of the Spearman rank correlation coefficients between $DI_{1}$ and the centrality parameters revealed similar results compared to Figure 4 and are, thus, not illustrated. These results are in accordance with the correlation coefficients obtained for $DI_{1}$ and $DI_{2}$ which are already explained above.

### 3.3. Rank Differences between Subsequent Rank Positions for the Winner-Loser Networks

**Figure 5:**
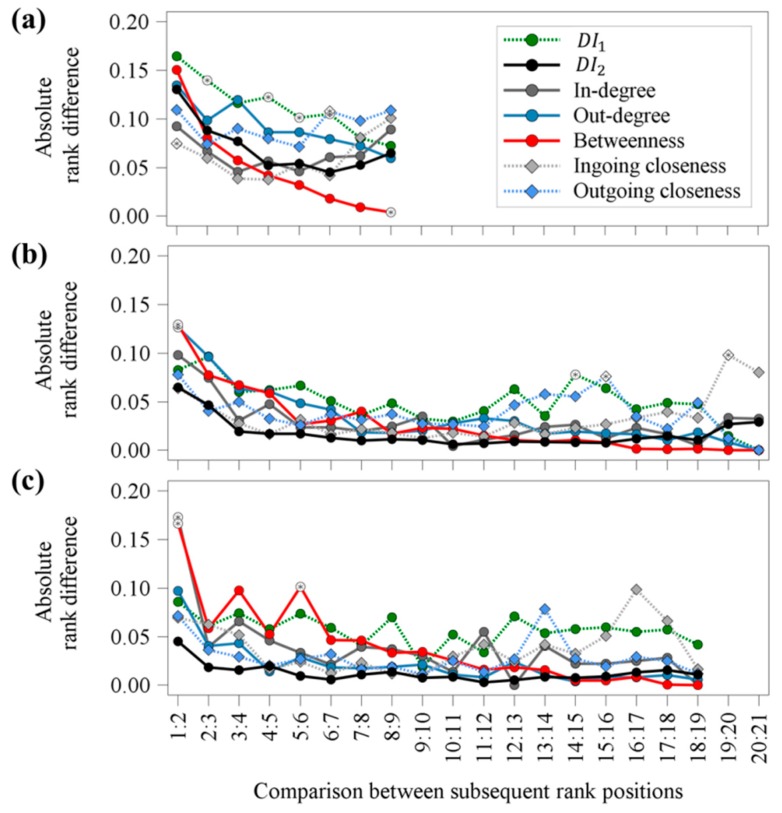
Absolute rank differences between subsequent rank positions for the parameters $DI_{1},\;DI_{2}$, in-degree, out-degree, betweenness, ingoing closeness and outgoing closeness for (**a**) weaned piglets, (**b**) fattening pigs and (**c**) gilts regarding the winner-loser networks after 17 h of video observation. Significant differences (*p* < 0.05) between $DI_{2}$ and the other parameters are illustrated with white filled markers containing a black asterisk.

The results of the absolute rank differences between subsequent rank positions revealed a significant impact of the interaction between parameter and analyzed comparison (Figure 5). Small rank differences with values below 0.2 were detected for all three age groups. Although the rank differences between all parameters were small, significant differences between $DI_{2}$ and the other parameters were detected. For weaned piglets, more significant differences were observed compared to fattening pigs and gilts. However, no clear pattern or specific parameters showing a significant difference were obtained for all three observed age groups. Furthermore, the results revealed higher rank differences between top-ranked animals compared to lower-ranking animals. This was apparent for almost all calculated parameters and all considered age groups.

### 3.4. Partial Least Squares Structural Equation Modelling (PLS-SEM)

**Figure 6:**
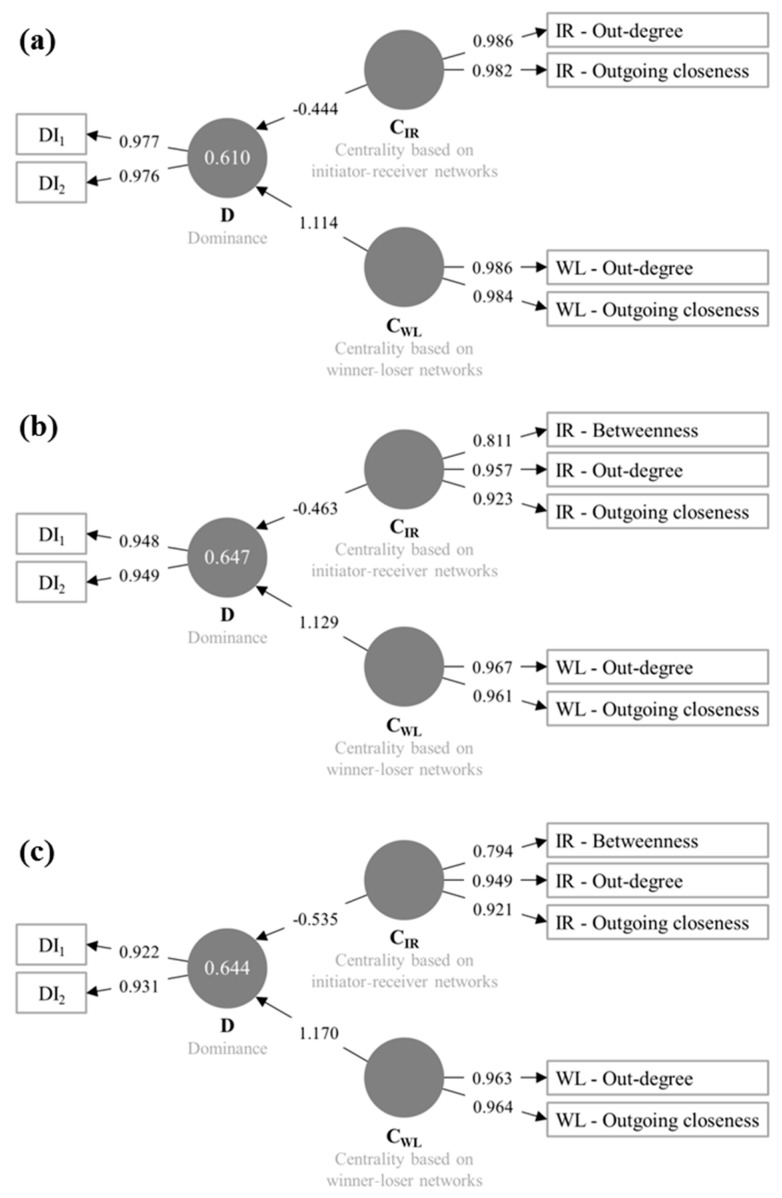
Calculated partial least squares structural equation modelling (PLS-SEM) for the relationship between dominance and centrality for the three age groups ((**a**) weaned piglets, (**b**) fattening pigs, (**c**) gilts). Circles represent latent variables; rectangles represent measurement variables. Arrows between circles represent relationships between latent variables (path coefficients). Arrows between circles and rectangles represent indicator reliabilities (of the respective measurement variables). Numbers in circles represent the coefficient of determination (which is only calculated in case of endogenous variables). IR indicates the centrality parameters calculated for the initiator-receiver networks. WL indicates the centrality parameters calculated for the winner-loser networks.

The PLS-SEMs for all three age groups contained the endogenous latent variable dominance (D) and the two exogenous latent variables centralities obtained from initiator-receiver networks (C_IR_) and centralities obtained from winner-loser networks (C_WL_)) (Figure 6). All models reached the goodness-of-fit criteria described in the materials and methods section. In total, with both exogenous variables (C_IR_ and C_WL_), about 60% of D can be explained in all age groups (weaned piglets: 61%, fattening pigs and gilts: 64%). 

The path coefficients were in all models statistically significant (*p* < 0.05). For weaned piglets, the path coefficient of C_WL_ with 1.114 was clearly higher compared to the path coefficient of C_IR_ with −0.444, similar to the results for fattening pigs and gilts.

The two measurement variables for D, $DI_{1}$ and $DI_{2}$, revealed the highest indicator reliabilities for weaned piglets, followed by fattening pigs and gilts, however all above 0.9. The PLS-SEMs differed slightly between the three age groups. The measurement variables for both exogenous latent variables, C_IR_ and C_WL_, out-degree and outgoing closeness were included in all models, which showed the highest indicator reliabilities with values above 0.9 in all models. For fattening pigs and gilts, betweenness calculated for the initiator-receiver networks was included in the model with indicator reliabilities of 0.811 and 0.794 for C_IR_.

## 4. Discussion

This study investigated whether centrality parameters derived from social network analysis are able to capture the rank order calculated by classical dominance indices. For this purpose, two different dominance indices, as well as five different centrality parameters based on two network types (initiator-receiver networks and winner-loser networks), were calculated regarding agonistic interactions observed in three rehousing and mixing events (weaned piglets, fattening pigs, gilts). In addition to the isolated consideration of the relation between each single dominance index and centrality parameter with the help of Spearman rank correlations and the individual rank differences, a more holistic approach using the partial least squares structural equation modelling (PLS-SEM) was carried out which enabled a joint analysis of all calculated parameters.

The results of the present study demonstrate that for the three observed age groups no substantial differences for the correlation between the dominance indices and the centrality parameters were obtained. Also, the choice of the dominance index calculated has no substantial impact. Although Langbein and Puppe [21] stated that $DI_{1}$ is only valid if the data set contains few unknown dyads and if all individuals of the group participate in a similar number of agonistic interactions. In the present study, this was definitely not the case in the first few hours analyzed for all age groups. Here, indeed, the number of isolated nodes decreased within the first 6 h after rehousing and mixing. Nevertheless, in these first few hours, the number of unknown dyads was still very high. However, looking at the results of the correlations between $DI_{1}$ and all centrality parameters similar values compared to the correlations between $DI_{2}$ and all centrality parameters were obtained. Furthermore, although the frequency distribution of both dominance indices differed clearly from each other, the correlations between both calculated dominance indices were always very high ranging from 0.85 to 0.92. It should be noted that $DI_{2}$ delivers more precise results considering individual rank differences between the animals compared to $DI_{1}$ due to the inclusion of the number of opponents won or lost. This results in a narrower frequency distribution for $DI_{2}$ resulting in a smaller range of values compared to $DI_{1}$. These results were also confirmed by the PLS-SEM which showed high indicator reliabilities for both dominance indices in all age groups. Thus, in the present study, the choice of the dominance index was not that important, but the choice of network type and centrality parameter, which should be compared to the dominance indices, has more impact on the presented results, which are discussed in the next two sections.

### 4.1. Choice of Network Type

In the present study, two types of networks were created in order to evaluate their impact on the relation between dominance indices and centrality parameters. The results show that independent of the dominance index used, as explained above, the winner-loser networks demonstrate better distinguishable results with a larger range of $r_{s}$ values compared to the initiator-receiver networks. This could also be confirmed by the results of the PLS-SEM. Here, the higher path coefficients for the centrality parameters based on the winner-loser networks (C_WL_) indicate that these values are more suitable to measure dominance compared to the centrality obtained from initiator-receiver networks (C_IR_). This can be explained by the fact that the calculated dominance indices are also based on information based on won and lost fights, so the results for the winner-loser networks resembled the dominance index more compared to the initiator-receiver networks. This is also confirmed by the number of initiated fights which resulted in a victory for the initiator. Here, only about two thirds (weaned piglets and fattening pigs) or half (gilts) of the initiated fights resulted in a victory for the initiator. However, we decided to also include the initiator-receiver networks due to the fact that the detection of the beginning of a specific behavioral pattern is less time-consuming than the detection of its end and its outcome. When working with the already time-consuming and lavish video analysis, it is of special importance to identify ways to reduce the workload and to increase the efficiency of this technique. 

Another explanation for the better agreement between the winner-loser networks and the dominance indices is the fact that, in the present study, the ethogram defining agonistic interactions were based on overt agonistic interactions including physical contact between the two fighting animals. Other studies have shown that in older age groups with more experienced and mature animals more subtle displays of dominance behavior or threats are used in order to clarify the social hierarchy within the group [20,37,38]. However, the inclusion of these behavioral patterns (e.g., threats) would immensely increase the complexity and the error rate of the video analysis due to the less overt character of these behavioral patterns. Also, McGlone [22] stated that threats are usually too subtle for the human observer and are thus difficult to measure objectively. However, if these behavioral patterns are included in the analysis, it might also improve the results for the initiator-receiver networks, which cannot be answered with the results obtained from the present study. Here, based on the ethogram used, the results of the winner-loser networks were better able to capture the rank order calculated by dominance indices.

### 4.2. Choice of Centrality Parameters

For the winner-loser networks, the correlations between the dominance indices and the centrality parameters revealed three clearly distinguishable groups for all age groups considered. Highly positive $r_{s}$ values were obtained for the correlations between the dominance indices and out-degree as well as outgoing closeness. Moderately negative $r_{s}$ values were obtained for the correlations between the dominance indices and in-degree as well as ingoing closeness. Low to moderately positive $r_{s}$ values were obtained for the correlations between dominance indices and betweenness. This relation was also present in the initiator-receiver networks. However, here, the correlation coefficients between the dominance indices and the three parameters out-degree, outgoing closeness and betweenness were nearly identical. Furthermore, the results of the PLS-SEM show similar results compared to the $r_{s}$ values in all observed age groups. The centrality parameters based on outgoing edges, out-degree and outgoing closeness, which had the highest $r_{s}$ values over the whole observation period, also revealed good measurements for the latent exogenous variables in the model with indicator reliabilities above 0.9. One possible explanation for these results could be that out-degree and outgoing closeness reflect active behavior (e.g., initiation of an agonistic interaction, being dominant), whereas in-degree and ingoing closeness reflect more passive behavior (e.g., being attacked, being subordinate). This relation becomes even more important when the social structure of farm animals is analyzed. The natural behavior of the animals is influenced by the predefined housing conditions, i.e., limited available space, low means of escape, predefined pen mates, restricted feeding [4]. Particularly, the restricted possibility for subordinate animals to avoid more dominant pen mates leads to an increased level of agonistic interactions in conventional husbandry systems [12]. 

### 4.3. Advantages of Social Network Analysis

Although social network analysis provides a complete picture of the individual nodes of the network as well as the entire network structure including indirect connections, its application in studies of farm animals is underrepresented. For the analysis of agonistic interactions, the indirect connections especially can have an immense impact on network structure. They can be seen as cascade initiated by one agonistic interaction [39,40], i.e., the initial agonistic interaction between animals A and B can cause another agonistic interaction between animals B and C. Thus, the first direct interaction between animals A and B indirectly affects the interaction of animal C, although animals A and C did not directly interact with each other [40]. Furthermore, with regard to dominance hierarchies, the social network approach has some advantages compared to the conventional methodologies. According to Klass et al. [41], the presence of unknown relationships can affect conventional analysis of dominance. Furthermore, Douglas et al. [42] stated that the network approach is not limited by the minimum or the maximum number of the study population as this is the case in conventional analyses of dominance. Another advantage of social network analysis is that the centrality parameters can be used without the underlying assumption of linearity compared to the calculation of dominance indices [42,43]. Furthermore, the present study revealed also low-rank differences between subsequent rank positions for all calculated parameters. This indicates that not only the rank order obtained for dominance indices and centrality parameters was similar, but also the individual rank differences demonstrated comparable results. Here, it should be noted that the results of the rank differences revealed higher values for the top-ranking animals compared to lower-ranking animals. This indicates that in all age groups and for almost all calculated parameters the most dominant animals were clearly distinguishable from the animal at the subsequent rank position. These findings could also be confirmed by Meese and Ewbank [17] who analyzed the relationship between social rank and being the most aggressive pig in the group. The results revealed that the difference in the number of incidents of aggression for subsequent rank positions was also higher for the top-ranking animals compared to lower-ranking animals. In the study of Foister et al. [6], social network properties were used in order to predict chronic aggression in commercial pig systems. The results revealed that network properties (e.g., large clique formation, betweenness centralization) calculated for data 24 h after mixing could better predict long term aggression (3 weeks after mixing) than simple dyadic traits. Per definition, all animals which are part of the same clique fight with each other. Thus, clique members may form better-established dominance relationships than animals that do not belong to the same clique. Moreover, the results of the study of Foister et al. [6] demonstrated that the clique sizes did not exceed 50% of the animals in each pen, i.e., a central group with an established dominance hierarchy is sufficient to reduce lesions caused by agonistic interactions at pen level, whereby not all animals have to be involved directly. Here, the indirect connections illustrated by the social network analysis become important. Thus, network properties can be used for the identification of high-risk groups for deciding which specific intervention strategies can be implemented. This is also confirmed by Sih et al. [44], who stated that it is not only important to know the direct social interactions of an animal, but also the indirect ones, i.e., to know with which other animals one particular animal’s social partners interact. For dominance hierarchies, such behavioral cascades can immensely impact the social structure of a group, i.e., animals with a high rank may have a positive effect on low ranking animals by suppressing individuals of intermediate rank. Furthermore, Boyland et al. [13] stated that low network stability can be used as an indicator of social stress, which has implications for animal welfare and productivity. 

### 4.4. Limitations of the Study

One limitation of the present study was that no direct comparison between the three age groups was possible or only possible to a limited extent due to the confounding factors age, level of familiarity as well as group size. Previous studies have revealed that younger animals demonstrated more agonistic interactions after mixing with a higher content of vigorous fighting compared to older animals [16,45]. Adult animals gained more experience from previous agonistic interactions and can thus better evaluate their own as well as the fighting ability of their pen mates [45]. Also, the higher level of familiarity in the two older age groups may have influenced the present results, which was, however, inevitable due to the on-farm conditions. It is known that acquainted animals show fewer agonistic interactions due to the fact that the rank positions are already clarified from previous mixing events [46,47]. The last confounding factor is group size. Several studies have stated that animals in smaller groups tended to have more agonistic interactions than animals in larger groups [48,49,50,51]. These findings could also be confirmed by the present study. Especially the group size is also known to have an impact on the results of social network analysis. In groups with a higher number of nodes, the probability to form an edge between each pair of animals is lower compared to a smaller group. Furthermore, it should also be mentioned that for the calculations of the dominance indices, the linearity of the hierarchy is assumed, otherwise, the dominance indices would not give meaningful results. This was not explicitly tested in the present study. Although it is often stated that larger groups of pigs tend to have more complex relationships and are less transitive [52,53], also in larger groups a semi-linear hierarchy was found: 20 sows [54]. However, rank instabilities within the middle and lower part of the hierarchies could be observed [54,55]. Thus, dominance indices calculated in groups without a strict linear hierarchy might not be displayed precisely and should be interpreted with caution.

## 5. Conclusions

The highly positive $r_{s}$ values between the dominance indices and the centrality parameters out-degree and outgoing closeness, the low-rank differences between subsequent rank positions as well as the results of the PLS-SEMs over all analyzed age groups illustrate that out-degree and outgoing closeness are able to capture the rank order obtained from dominance indices. However, this statement holds only for winner-loser networks and not for initiator-receiver networks. Thus, a detailed analysis of the outcome of each agonistic interaction is necessary in order to obtain a similar rank order with the above-named centrality parameters in comparison to the dominance indices. Future studies can determine the dominance hierarchy within a group using centrality parameters and profit from further parameters derived from a social network analysis which provides more detailed insights into group structure, which goes beyond the simple dyadic approach provided by dominance indices.

## Figures and Tables

**Table 1:** Basic information about the study design, housing conditions, involved animals and observation periods.

Parameter	Weaned Piglets	Fattening Pigs	Gilts
Age at time of mixing	26 days	68 days	154 days
Level of familiarity ^1^	0 animals	2 animals	5 animals
Compartment	Flatdeck	Fattening unit	Breeding unit
Dimensions	2.05 m × 1.36 m	3.25 m × 2.40 m	7.20 m × 5.40 m
Floor	Concrete & metal	Half-slatted & -solid	Half-slatted & -solid
Feed	Solid pelleted feed	Mash feeding machine	Mash feeding machine
Water	Nipple drinkers	Nipple drinkers	Nipple drinkers
Number of animals	829	543	249
Number of pens	93	26	12
Number of animals/pen	8.9 ± 0.6 (6–11)	20.9 ± 1.7 (17–25)	20.8 ± 3.4 (16–27)
Average male:female ratio	1:1.14	1:1	-
Video observation	28 h	17 h	17 h
Observation periods	Day 1: 12:00–18:00	Day 1: 12:00–18:00	Day 1: 12:00–18:00
	Day 2: 07:00–18:00	Day 2: 07:00–18:00	Day 2: 07:00–18:00
	Day 3: 07:00–18:00	-	-

^1^ Maximum number of animals in a pen already acquainted with each other.

**Table 2:** Description of the calculated centrality parameters.

Parameter	Description
In- & Out-degree	Number of ingoing (in-degree) or outgoing (out-degree) interactions. In the initiator-receiver networks, in-degree describes the number of received fights and out-degree describes the number of initiated fights. In the winner-loser networks, in-degree describes the number of fights lost and out-degree describes the number of fights won [8].
Betweenness	Measures the extent to which an animal lies on shortest paths to other animals [9,28].
Ingoing & Outgoing closeness	Mean distance from all reachable animals to any other specific animal (ingoing closeness) or mean distance from one specific animal to all other reachable animals (outgoing closeness) [9,29,30].
